# Molecular diagnostics in neurotrauma: Are there reliable biomarkers and effective methods for their detection?

**DOI:** 10.3389/fmolb.2022.1017916

**Published:** 2022-09-29

**Authors:** Davran Sabirov, Sergei Ogurcov, Irina Baichurina, Nataliya Blatt, Albert Rizvanov, Yana Mukhamedshina

**Affiliations:** ^1^ Institute of Fundamental Medicine and Biology, Kazan Federal University, Kazan, Russia; ^2^ Neurosurgical Department No. 2, Republic Clinical Hospital, Kazan, Russia; ^3^ Department of Histology, Cytology, and Embryology, Kazan State Medical University, Kazan, Russia

**Keywords:** biomarkers, traumatic brain injury, spinal cord injury, enzyme-linked immunosorbent assay, proteomic analysis, transcriptomic analysis, metabolic analysis

## Abstract

To date, a large number of studies are being carried out in the field of neurotrauma, researchers not only establish the molecular mechanisms of the course of the disorders, but are also involved in the search for effective biomarkers for early prediction of the outcome and therapeutic intervention. Particular attention is paid to traumatic brain injury and spinal cord injury, due to the complex cascade of reactions in primary and secondary injury that affect pathophysiological processes and regenerative potential of the central nervous system. Despite a wide range of methods available methods to study biomarkers that correlate with the severity and degree of recovery in traumatic brain injury and spinal cord injury, development of reliable test systems for clinical use continues. In this review, we evaluate the results of recent studies looking for various molecules acting as biomarkers in the abovementioned neurotrauma. We also summarize the current knowledge of new methods for studying biological molecules, analyzing their sensitivity and limitations, as well as reproducibility of results. In this review, we also highlight the importance of developing reliable and reproducible protocols to identify diagnostic and prognostic biomolecules.

## Introduction

Neurotrauma is a serious public health problem worldwide due to the high disability of a young working-age population and the high cost of medical support (DeVivo, 2012; Rubiano et al., 2015; Asmamaw et al., 2019). The problem of functional recovery of patients with traumatic brain injury (TBI) and spinal cord injury (SCI) is especially relevant due to low regenerative potential of the central nervous system (CNS). Primary neurotrauma, leading to cell necrosis in the area of a traumatic force application, is replaced by secondary, even more catastrophic damage to the nervous tissue. A complex cascade of inflammatory, toxic and vascular reactions causes the death of neurons and glial cells, accompanying progressive structural changes in brain and spinal cord tissues (Gaudet and Fonken, 2018; Orr and Gensel, 2018).

However, the severity of posttraumatic reactions and the intrinsic regenerative potential of the nervous tissue may be different for individual subjects, which may determine the degree of damage and functional outcomes of the subacute period of neurotrauma. Sometimes it is difficult to establish the severity of the injury applying neuroimaging methods used in the clinic (MRI, CT) (Amyot et al., 2015; Dalkilic et al., 2017). With the help of a functional neurological examination, the clinical assessment of the severity by American Spinal Injury Association (ASIA) Impairment Scale (AIS) grade and the Glasgow Outcome Scale (GOS)/Glasgow Coma Scale (GCS) in SCI and TBI accordingly is of great importance (Weir et al., 2012; Betz et al., 2019). However, the baseline clinical examination is often difficult to perform during the acute period of neurotrauma, and the variability in spontaneous recovery within each grade of AIS and GOSE is very high (Sandwell and Markandaya, 2015).

At the same time, quantitative and qualitative changes in the molecular composition of cerebrospinal fluid (CSF) and blood serum caused by cell damage or an increased permeability of cell membranes and, in general, violations of the blood-brain barrier can make it possible to detect differences in the severity of pathophysiological processes after TBI and SCI (Halford et al., 2017). Given the above, the identification of reliable biomarkers in these biological fluids that can provide an objective and accurate diagnosis of neurotrauma, predict functional outcomes and, in particular, monitor the effectiveness of therapy, may be of decisive importance in medical support (Capirossi et al., 2020).

To date, there is a sufficient number of studies that search for biomarkers of neurotrauma of varying severity. To this end, researchers use various methods and approaches that differ in time, complexity, and cost. However, the clinic still does not have access to a test system that has the ability to accurately and easily diagnose and predict damage to the brain and spinal cord. This review is aimed at describing potential biomarkers correlated with the degree of neurotrauma, with a focus on the sensitivity and objectivity of the methods used and the reproducibility of the results.

## Enzyme-linked immunosorbent assay

Enzyme-linked immunosorbent assay (ELISA) has been long known to be used to search for neurotrauma biomarkers. However, the earliest works primarily focused on etiopathogenesis, and only subsequently made a conclusion about the possibility of diagnostic, prognostic and therapeutic significance of the obtained data (Segal and Brunnemann, 1993; Wang et al., 1995; Segal et al., 1997; Kil et al., 1999). Later works, dating back to the beginning of the 2000s according to the sources known to us, already preferred to identify clinical correlates of elevated blood serum/CSF cytokines/autoantibody or other proteins in patients with neurotrauma (Fassbender et al., 2000; Pleines et al., 2001; Hayes et al., 2002; Franz et al., 2003).

Since 2018, the U.S. Food and Drug Administration (FDA) has approved marketing of a first rapid ELISA to assess mild TBI by serum glial fibrillary acidic protein (GFAP) and ubiquitin carboxy-terminal hydrolase L1 (UCH-L1) levels. Using the abovementioned Banyan BTI™ test Lewis et al. (2020) showed that in the first 6 h after TBI the median serum concentrations of GFAP and UCH-L1 were significantly higher in patients with acute unfavorable neurological outcome detected by computer tomography (CT^+^). However, it should be noted that UCH-L1 and GFAP level did not show any significant association with an outcome 3 months after injury (Diaz-Arrastia et al., 2014). Levels of GFAP and UCH-L1 expressions are also being actively studied in SCI (Kwon et al., 2010, 2017; Yokobori et al., 2015). The results of these studies are consistent and indicate a positive correlation between CSF levels of GFAP and UCH-L1 in the acute period of SCI and the severity of damage.

The standard ELISA method has low sensitivity and is poorly suited for searching biomarkers of neurotrauma due to the low circulating concentration of the brain-specific proteins in blood, since the blood-brain barrier limits their diffusion (Marchi et al., 2003). In this regard, new methods for conducting ELISA are being developed (Li et al., 2016; Hu et al., 2020; He et al., 2021). O’Connell et al. (2020) in attempt to determine the advantage of digital ELISA over conventional ELISA methods compared the levels of neuron-specific proteins in TBI patients no more than 24 h after injury. The authors showed that digital ELISA measures of neurofilament light chain (NF-L) and tau protein had a greater diagnostic efficiency and 100% sensitivity compared to the 7.7% in those estimated by conventional ELISA (O’Connell et al., 2020).

Czeiter et al. (2020) assessed levels of 6 serum biomarkers in TBI, two of which -S100B and NSE- were measured using an electrochemiluminescence immunoassay, which shows higher sensitivity, reduction matrix effects, and requires lower sample volumes (Bolton et al., 2020). The study found that the abovementioned biomarkers correlated with the trauma-related intracranial findings on CT and the requirement for hospitalization in a general ward or intensive care unit. However, it is worth noting that the half-life of S100B is ∼90 min, in contrast to NSE, which has a half-life of ∼24 h, thus reducing the clinical utility of S100B as a neurotrauma biomarker (Ghanem et al., 2001; Kang et al., 2021).

Over the past decade, many studies have been conducted using traditional or improved ELISA in search for and quantitative assesment of neurotrauma biomarkers (Table 1). Most of the ELISA kits used are not designed or approved for human use and therefore are not regulated, often leading to a marked variability of results over time among test kits and different laboratories using them (Feng et al., 2019). In addition, there is an understanding that for more informative data it is necessary to obtain results for several biomarkers. Simultaneous measurement of multiple biomarkers with ELISA when run in parallel is time consuming, increases the risk of errors and requires large sample costs often collected in small volume due to their value (Ray et al., 2005). In this regard, the development of multiplex immunoassay technologies, discussed below, made for solving some of the problems and brought the research related molecular diagnostics in neurotrauma to a new level.

**TABLE 1:** Candidate biomarkers that are detected by immunoanalysis in neurotrauma.

Biomarker candidates	Period of neurotrauma	Samples	Correlation (/) with clinical characteristics	Neurotrauma (key ref.)
ELISA				
GFAP	Acute	Blood serum	/with outcome	TBI: Lewis et al. (2020)
		CSF	/with injury severity	SCI: Ahadi et al. (2015)
			/with injury severity and outcome	Yokobori et al. (2015)
				Kwon et al. (2010, Kwon et al., 2017)
GFAP-BDP	Acute	Blood serum	/with injury severity and outcome	TBI: Okonkwo et al. (2013)
UCH-L1	Acute	Blood serum	/with outcome	TBI: Lewis et al. (2020)
		CSF	/with injury severity	SCI: Yokobori et al. (2015)
S100b	Acute	CSF	/with injury severity and outcome without/with outcome	SCI: Kwon et al. (2010, 2017)
	Acute/subacute	Blood serum/CSF		TBI: Shahim et al. (2016)
tau	Acute	CSF	/with injury severity and outcome	SCI: Kwon et al. (2010, 2017)
NF-L, s-NF-L or pNF-H	Acute/subacute	Blood serum/CSF	/with injury severity and outcome	TBI: Shahim et al. (2016)
			/with outcome	Al Nimer et al. (2016)
	Acute	Blood serum	/with injury severity	SCI: Ahadi et al. (2015)
NSE	Acute	Blood serum	/with injury severity	SCI: Ahadi et al. (2015)
	Subacute			Ogurcov et al. (2021)
VEGF	Subacute	Blood serum	/with injury severity	SCI: Ogurcov et al. (2021)
Multiplex immunoassay				
Bio-plex Pro Human Cytokine 21-Plex (Bio-Rad)	Chronic	Plasma	N/D	SCI: Stein et al. (2013)
Human 25-plex kit (Invitrogen)	Acute	CSF	IL-6/with injury severity	SCI: Kwon et al. (2017)
			IL-6, IL-8, MCP-1/with outcome	
Human Neurology 4-Plex B (Quanterix)	Acute	Blood serum	GFAP/with CT abnormalities	TBI: Czeiter et al. (2020); Gill et al. (2018); Korley et al. (2019); Thelin et al. (2019)
Bio-Plex ProTM Human Cytokine 40-plex Assay (Bio-Rad)	Subacute	Blood serum	CXCL5, TNFα, CCL11, CXCL11, IL10, MIF/with injury severity	SCI: Ogurcov et al. (2021)

N/D, not detected. The table shows data from studies published since 2010. CSF, Cerebrospinal fluid; CT, Computed tomography; TBI, Traumatic brain injury; SCI, Spinal cord injury.

## Multiplex immunoassay

It was previously suggested that the development of a multiplex immunoassay to measure biomarkers of neurotrauma could be an important step in advancing research, as it would allow a faster and cheaper detection of required proteins in the smaller sample volumes (Berger et al., 2009). However, despite these advantages, this technology has not yet been introduced into clinical practice and so far, remains in demand only at the stage of fundamental research of new biomarkers and therapeutic targets.

Korley et al. (2019) measured GFAP, UCH-L1, NF-L, and total tau levels in patient plasma samples up to 24 h after TBI using an ultrasensitive 4-plex immunoassay. GFAP, NF-L and UCH-L1 values correlated with the detection of traumatic intracranial pathology on CT. Of a particular interest was the data showing that the sensitivity of NF-L measurement using the multiplex immunoassay kit was comparable to that of a singleplex analysis of similar samples (Korley et al., 2019). Many researchers showed interest in the abovementioned multiplex immunoassay kit for registering neurology biomarkers. The previously mentioned study by Czeiter et al. (2020) confirmed the correlation of GFAP, UCH-L1, NF-L and total tau levels with TBI severity. The most pronounced changes were found in the expression of GFAP, which level achieved the highest discrimination for predicting CT abnormalities within 24 h after TBI (Czeiter et al., 2020). However, a comprehensive analysis of GFAP in conjunction with the abovementioned proteins did not provide additional value for predicting CT^+^, confirming the data of previous works (Gill et al., 2018).

Stein et al. (2013) conducted a pilot study to simultaneously assess the level of 21 plasma cytokines of chronic SCI patients using multiplex immunoassay. It was found that there was a significant increase in the levels of MIF, CXCL9, MCSF, IL-3 and SCGF-β in chronic SCI patients compared to uninjured control, nevertheless, no correlation was found with any clinical characteristics. In another study, a 25-plex immunoassay kit was used to search for CSF biomarkers to stratify SCI severity and predict an outcome (Kwon et al., 2017). At 24 h post-injury the level of IL-6 was significantly different between patients with baseline AIS grades of A to C, and changes of IL-6, IL-8 and MCP-1 levels correlated with improvement of AIS grade over 6 months. Our recent pilot study, using extended multiplex analysis of 40 serum analytes of patients at 2 weeks post-SCI showed a large elevation of IFNγ (>52 fold), CCL27 (>13 fold), and CCL26 (>8 fold) (Ogurcov et al., 2021). At the same time the levels of cytokines CXCL5, CCL11, CXCL11, IL10, TNFα, and MIF were different between patients with baseline AIS grades of A or B.

Unfortunately, the low analytical sensitivity of multiplex immunoassay has not yet made it possible to make a significant breakthrough in molecular diagnostics of neurotrauma, moreover, this method is not cheap (Korley et al., 2019). Multiplex immunoassays often reduce analytical sensitivity due to interference between different antibodies, analytes, and assay diluents; variability of the production process; and incompatibility between different limits of quantitation (Ellington et al., 2009). Nevertheless, subsequent work to improve the analytical characteristics of multiplex immunoassay should contribute to its wider introduction into clinical practice, acquiring not only high diagnostic, but also a practical importance for correcting the therapeutic intervention plan.

## Proteomic analysis

**FIGURE 1:**
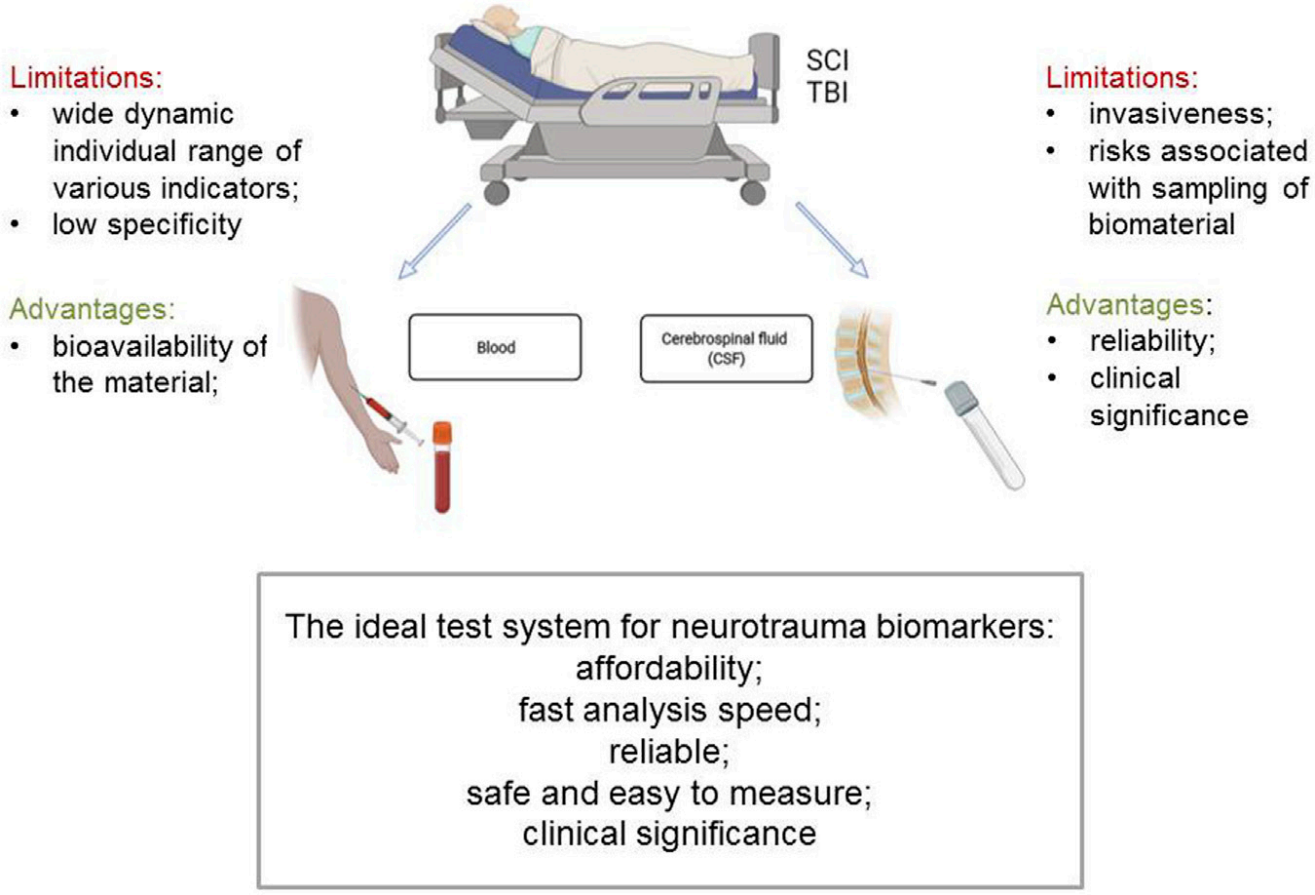
Possibilities of using blood and cerebrospinal fluid as biomarkers of neurotrauma.

Neurotrauma leads to a change in synthesis and secretion of many proteins, associated with the complexity and dynamism of posttraumatic processes. In this regard, the use of quantitative proteomics, which helps to recognize differentially expressed proteins in TBI and SCI, can not only identify potential diagnostic biomarkers, but also predict the goals and mechanisms of treatment (Zhou et al., 2020). For research purposes large-scale proteomics is most often used to identify posttraumatic changes in the qualitative and quantitative composition of proteins in CSF and blood serum (Poulos et al., 2020) (Figure 1). Moghieb et al. (2016) initially assessed changes in protein expression in injured rat spinal cord tissue and then determined whether these changes were reflected in CSF or blood serum. During the study, 12 proteins were identified as biomarkers of SCI, of which only transferrin, triosephosphate isomerase 1, cathepsin D, and astrocytic phosphoprotein PEA-15 were found to be elevated in CSF in both rodents and humans with SCI 24 h and 7 days after damage (Moghieb et al., 2016). Similar approach was taken by Skinnider et al. (2021) who evaluated the expression of 491 proteins in CSF and blood serum from patients with acute SCI and, in parallel, in a porcine model of SCI. The authors showed that level of GFAP in CSF correlated with both the initial severity of injury and more adverse neurological outcomes in both humans and pigs (Skinnider et al., 2021).

Haqqani et al. (2007) applied gel-free proteomic approaches to identify peripheral “surrogate markers” in serum blood samples obtained from children during the acute period of severe TBI. The authors found an increase in the level of proteins such as S100β, neuron-specific gamma-enolase, amyloid beta (A4) precursor protein, alpha-spectrin, and cleaved tau protein, which confirms previously obtained data (Olsson et al., 2004; Ringger et al., 2004). Based on proteomic analysis of blood serum within the first 24 h Anada et al. (2018) proposed a panel of 10 biomarkers for diagnosing patients with TBI of different severity which may be complementary to the Glasgow Coma Scale (GCS) system. The authors found that the level of kininogen, apolipoprotein E and zinc-alpha-2-glycoprotein can be used as protein signatures to differentiate the injury severity (Anada et al., 2018).

It should be noted that blood has the highest priority as a source of biomarkers, since sampling procedure has a low degree of invasiveness, does not lead to significant risks and does not require additional manipulations. However, the key problem is that the blood serum proteome reflects the collective expression of all tissues and cell types of the body and, accordingly, does not have a high specificity for nervous tissue; in addition, the problem also lies in the high dynamic range of proteins and peptides (Azar et al., 2017).

In order to identify biomarkers of neurotrauma using proteomic analysis, it is advisable to sample cerebrospinal fluid, because CSF maintains direct contact with the CNS. CSF has a unique advantage over plasma, saliva, and other fluid sources in its ability to reflect the biochemical changes that occur during neurotrauma. Thus it is widely used in proteomic analysis for the determination of biomarkers in TBI. Among 484 identified proteins Halford et al. (2017) found 232 unique enzymes for the acute period of severe TBI. Proteins associated with astrocyte injury (ALDOC, GLNA, BLBP and PEA15) were identified and confirmed for presence and quantification using two independent approaches: Immunoblotting with scaled densitometry and multiple reaction monitoring-mass spectrometry (MRM-MS). Comparison of the two methods showed similar detection limits and interquartile ranges for ALDOC, BLBP, and GFAP, further confirming the trends obtained with both approaches. However, the authors noted a wide dynamic range of biomarker concentrations, reflecting the greater clinical heterogeneity of TBI (Halford et al., 2017). In other investigation, the two approaches were also used to identify TBI biomarkers in CSF obtained microvesicles/exosomes (MVs/Es) (Manek et al., 2018). Several known TBI biomarkers were found to be present, such as αII-spectrin and GFAP degradation products, UCH-L1 and GFAP at higher concentrations in CSF obtained MVs/Es after TBI compared to similar samples in non-TBI control patients.

In contrast to the abovementioned studies, Streijger et al. (2017) conducted a targeted proteomic analysis of CSF samples obtained from patients with acute SCI using MRM-MS. The authors identified 27 potential biomarkers of neurotrauma (baseline AIS A, B, or C), with triosephosphate isomerase having the strongest association with SCI severity. An earlier study by Sengupta et al. (2014) used difference gel electrophoresis and mass spectrometry (MS) to compare the CSF proteomic profile of patients at days 1–8 and 15–60 after SCI. The authors identified 8 proteins whose expression level depended on SCI severity (AIS A vs. AIS C, D). According to the results, these proteins were involved in various molecular pathways, including DNA repair, protein phosphorylation, tRNA transcription, iron transport, mRNA metabolism, immune response, and lipid and ATP catabolism, while their level profiles changed over time after SCI (Sengupta et al., 2014).

The active development of proteomic analysis methods plays an important role in the discovery of biomarkers in patients with neurotrauma. However, proteomic analysis requires careful sample preparation, which takes a long time, about several days. Preliminary application of large-scale proteomics and subsequent targeted MS or microarray-based methods, often in combination with gel electrophoresis of CSF samples and, to a lesser extent, blood serum of patients with SCI and TBI, allows more efficient search for potential biomarkers that can be used to predict clinical outcomes. However, one of the main limitations of proteomic analysis is the presence of potential age-related proteins, presented individually. In this regard, significant further work is required, since none of the potential biomarkers is ready for a routine use in clinic practice.

## Transcriptomic analysis

Signaling cascades of primary and secondary damage triggered by neurotrauma of the CNS contribute to the development of inflammatory reactions and cell death; however, the disclosure of molecular mechanisms is still limited. In this regard, the analysis of transcriptome changes during TBI and SCI can provide key insights into the mechanisms and pathways associated with these pathologies, which will be extremely useful for improving the effectiveness of regenerative therapy and pharmacological screening. Studies using RNA-seq technology provide good coverage of neurodegeneration processes in humans (Alzheimer’s disease, Parkinson’s disease) (Hossein-Nezhad et al., 2016; D’Erchia et al., 2017), while such studies in CNS neurotrauma are most often performed on animal models (Du et al., 2018; Zhou et al., 2019). Thus, studies of animal spinal cord transcriptome at various stages of TBI and SCI helped developing a system analysis program used to identify key determinants in global gene networks, immune response associated enriched groups of genes, cytokine/chemokine activity, the MHC protein complex, processing and antigen presentation, translation, ion channel activity and small GTPase-mediated signal transduction (Chen et al., 2013; Zhong et al., 2016; Meng et al., 2017; Shi et al., 2017; Wang Q. et al., 2019; Li et al., 2019). There are also studies that utilize the method of transcriptomic profiling of animal spinal cord to reveal regenerative mechanisms against the background of various therapy options use (Duan et al., 2015).

Michael et al. (2005) analyzed 1,200 genes in 7 patients with TBI, of which the expression 104 genes were differentially changed when compared with the control group of healthy people. The authors indicated that most often significant differences were observed for genes that control the regulation of transcription, intermediate and energy metabolism, signaling, and intercellular adhesion (Michael et al., 2005). Kyritsis et al. (2021) exmained global gene expression in peripheral blood leukocytes during the acute phase of SCI and identified 197 genes which expression changed after injury, including in direct relation to the severity of SCI. In a similar but earlier study, Wang F. et al. (2019) found that differential gene expression in patients with incomplete SCI with capacity to recover motor function was significantly enriched in the neurotrophin TRK receptor signaling pathway. At the same time, the greatest difference between the groups of patients with incomplete/complete SCI and healthy people was in the expression level of *EPHA4*, *CDK16*, *BAD*, *MAP2* Normal 2, *EGR* and *RHOB* genes (Wang F. et al., 2019).

In addition to the global gene expression, active targeted studies are being conducted on post-transcriptional regulators that affect gene expression. For example, it was recently established that long non-coding RNAs (lncRNAs) play an important role in a wide range of biological processes and are expressed, among other things, in CNS neurotrauma. Yang et al. (2019) studied the profile of lncRNAs and mRNA in human contusion tissue after TBI. Alterations in the expression of 99 lncRNAs and 63 mRNAs were found in the area of TBI compared to control samples (Yang et al., 2019). In the TBI group, the five were most significantly up-regulated and down-regulated lncRNAs, three of which played an important role in the immune system, representing peptides derived from extracellular proteins that are HLA class II beta chain paralogs (Chowdhary et al., 2015). Thus, the authors established a correlation between lncRNAs and mRNA and concluded that overexpressed lncRNAs were also involved in the pathological process of TBI (Yang et al., 2019).

Earlier studies showed that miRNAs downregulate many more targets than previously thought, thus helping to determine the expression of tissue-specific genes in humans (Lim et al., 2005). Therefore, miRNA profiling is often used, which, unlike mRNA, are more specific and accurate, and because of their size, are more stable in plasma, since they are predominantly located in exosomes. miRNAs are highly expressed in the CNS, can cross the blood-brain barrier, are stable in peripheral biofluids, and can provide information about brain damage. There are a lot of clinical studies on the identification of miRNAs in blood (plasma, serum) or CSF in TBI (Table 2), while similar studies in SCI are few (Lim et al., 2005; Tigchelaar et al., 2019).

**TABLE 2:** MiRNA biomarkers TBI in different biological sources from human.

TBI severity (GCS)	miRNA	Expression changes/period of TBI	Targets	Potential effects	References
CSF					
mild (9–15), moderate (9–15), severe (3–8)	miR-451	↑ 48 h	N/A	N/A	Bhomia et al. (2016)
	miR-328		EPO, EPOR	Mediators of erythropoietin signaling	
	miR-362-3p		SCN4a	Generation and propagation of neurons	
	miR-486		GABA	Receptor signaling	
severe (≤8)	miR-141, miR-572	↑ 14 days	N/A	N/A	You et al. (2016)
	miR-27b, miR-30b				
	miR-483-5p, miR-30b				
	miR-1289, miR-193b				
	miR-499-3p				
	miR-181a		N/A	Role in neuroinflammatory responses of astrocytes	
	miR-431		MTRNR2L1	Involved in the process of ischemia and reperfusion injury of cortical neurons	
	miR-1297, miR-33b	↓ 14 days	N/A	N/A	
	miR-933, miR-449b				
severe (≤8)	miR-451	↑ in period of venticular drainage	Dicer, FGFR1, CD133	Involved in erythropoiesis	Patz et al. (2013)
	miR-9	↓ in period of venticular drainage	N/A	Neuronal processes such as neuron development or axis formation	
Peripheral Blood					
severe (3–8)	miR-18a,	↑ 12 h	N/A	N/A	Ma et al. (2019)
	miR- let-7b	↑ 12–48 h	SERPINe, IL-6	anti-inflamatory role	
	miR-146a, miR-149	↑ 12,24,72 h	N/A	N/A	
	miR-203		MyD88	negatively regulates ischemia-induced microglia	
	miR-23b	↑12–72 h	N/A	decreasing lesion volume, alleviating brain edema, inhibiting neuronal apoptosis and attenuating long-term neurological deficits	
	miR-let-7f	↑ 24 h		survival and increased the production of cytokines	
	miR-181d, miR-29a	↑ 48 h		N/A	
	miR-18b				
	miR-199a-3p, miR-let-7a	↓ 24 h			
	miR-214	↓ 24–48 h			
Plasma					
severe (≤8)	miR-16	↓ 0–24 h	N/A	regulating cell proliferation, cell cycle progression, and apoptosis	Redell et al. (2010)
	miR-92a			negative regulator of angiogenesis	
	miR-765	↑ 0–24 h		N/A	
mild (>12)	miR-16	↑ 0–10 h		regulating cell proliferation, cell cycle progression, and apoptosis	
	miR-92a			negative regulator of angiogenesis	
mild (<13, 15)	miR-142-3p, miR-423-3p	↑ 24 h	N/A	N/A	Mitra et al. (2017)
mild (≥13), modarate (9–12), severe (≤8)	miR-3195, mir-328-5p	↑ 24 h	N/A	N/A	Qin et al. (2018)
	miR-6867-5p, miR-3665				
	miR-762				
	miR-4996, miR-2861				
mild (≥13), modarate (9–12), severe (≤8)	miR-940, miR-1281	↓ 24 h	N/A	N/A	Qin et al. (2018)
	miR-1825, miR-4665-3p				
	miR-4725-5p, miR-1304-3p				
Serum					
mild (9–15), moderate (9–15), severe (3–8)	miR-451, miR-505	↑ 48 h	N/A	N/A	Bhomia et al. (2016)
	miR-92a		ADRB1		
	miR-328		EPO, EPOR	Mediators of erythropoietin signaling	
	miR-151-5p, miR-326-3p		SCN4a	Generation and propagation of neurons	
	miR-20a, miR-30d		GABA	Receptor signaling	
	miR-195, miR-486				
mild (≥13)	miR-425-5p, miR-502	↓ 0–48 h	N/A	N/A	Di Pietro et al. (2017)
severe (≤8)	miR-21	↑ 4–72 h, 15 days			
severe (≤8)	miR-335	↑ 0–72 h, 15 days			
mild (13–15), moderate (9–12), severe (3–8)	miR-93	↑ 1–7 days	neurotrophin	Involved in the development of neural cells	Yang et al. (2016)
	miR-191		brain-derived neurotrophic factor	Modulates brain development and hippocampal neurogenesis	
	miR-499		N/A	Cell proliferation, apoptosis, the cell cycle, and cytoskeletal remodeling	
mild (13–15), moderate (9–12), severe (≤8)	miR-3610, miR-3907	↑ 1,7, 28 days; 5 years	N/A	N/A	Taheri et al. (2016)
	miR-126-3p	↑ 5 years			
	miR-126-3p	↓ 1, 7, 28 days			
Saliva					
mild (a concussion)	miR-142-3p,miR-135b-3p	↑ 48–72 h	N/A	N/A	Di Pietro et al. (2018)
	let-7i-5p			regulatory pathways of several inflammatory cytokines	
	miR-27b-3p		Bcl-2 (Noxa, Puma, Bax)	proapoptotic role	
	miR-107		granulin/progranulin	N/A	
mild (≤12)	miR-769-5p, miR-1307	↑ 4 weeks	N/A	N/A	Johnson et al. (2018)
	miR-133a-5p, let 7a-3p	↓ 4 weeks			
	miR-320c-1,			memory effect	
	miR-629			associated with headaches	
	let-7b-5p			associated with tired a lot	

N/A, not available; TBI, traumatic brain injury; GCS, Glasgow Coma Scale.

For example, Mitra et al. (2017) determined the level of miRNAs circulating in blood plasma on days 5 and 30 after TBI of various severity. miR-142-3p and miR-423-3p showed the highest potential clinical relevance for identifying mild TBI patients with post-concussion syndromes. In another study, miRNA expression in the serum of these patients was also measured to determine the differences between mild and severe TBI (Di Pietro et al., 2017, 2018). miR-425-5p and miR-21 have been shown to be reliable predictors of a favorable 6-month outcome in the first 12 h after mild TBI. miR-335 has been proposed as a promising biomarker for polytrauma-associated severe TBI. A year later, a similar study was published by Qin et al. (2018) in which the authors also assessed differences in the miRNA expression in blood plasma in the first 24 h after receiving mild, moderate, and severe TBI. In this study miR-3195 and miR-328-5p expression levels were higher in the severe TBI group than in the mild and moderate TBI groups (Qin et al., 2018). In addition to the blood serum miRNA, CSF miRNA was also studied within 48 h after TBI (Bhomia et al., 2016). Ten miRNAs, including miR-328, were found to be overexpressed by real-time PCR when compared TBI to healthy controls. The authors concluded that with an increase in the degree of damage detected on CT, more miRNAs are secreted into the serum, which is an indirect indicator of the severity of damage to the nervous tissue.

Early studies of miRNAs in SCI are based on the study of the miRNA profile of injured tissue in rat and mouse animal models (Liu et al., 2009; Strickland et al., 2011; Yunta et al., 2012; Baichurina et al., 2021). More recent studies by Tigchelaar et al., 2017 and Tigchelaar et al., 2019 are devoted to searching for miRNAs as biomarkers of SCI in blood serum and CSF of pigs and humans. The latest work on the assessment of the small RNAs profile, including miRNAs, in the acute patients (days 1 and 5) showed that, depending on the severity of damage, the concentrations of small RNAs increase in CSF in the first 24 h after neurotrauma, followed by a decrease in their concentration level on day 3 to the values observed in non-SCI control patients. It was found that miR-133 and miR-145-3p are statistically significantly increased in the serum of patients with SCI and also show severity-dependent expression. It should be noted that half of the miRNAs that were differentially expressed depending on the severity in blood serum of pigs also showed similar expression pattern in humans after SCI (Tigchelaar et al., 2017, 2019). However, the main cross-species difference was that in contrast to the experiments on pigs, in SCI patients the biggest change in the miRNA profile was observed in CSF, showing a correlation between the expression of certain serum miRNAs with injury severity and neurological outcome.

Transcriptomic analysis is expensive and takes a long time to complete. Nevertheless, the above examples indicate the prospect of using microRNA as a diagnostic tool. It is assumed that if one selects microRNAs that can characterize different degrees of neurotrauma, one can create an effective panel for accurate diagnosis. Unfortunately, studying the profile of microRNAs in biological fluids is a rather difficult task, which is associated with their low concentration and the lack of standard approaches for the isolation and analysis of miRNAs. In addition, one of the limitations of serum or plasma miRNA studies is the difficulty in determining the origin of a particular miRNA and identifying its effects.

## Metabolic analysis

Metabolomics is defined as a method for identifying metabolites synthesized by biological and physiological systems and is a phenotypic expression of genome and proteome. The use of high-throughput metabolomics methods may be useful in discovering new biomarkers associated with homeostasis disorders after neurotrauma (Wolahan et al., 2016).

Glenn et al. (2013) examined CSF of patients with severe and mild TBI in acute period (24 h) using proton nuclear magnetic resonance spectroscopic analysis. The study showed that the concentration lactate, propylene glycol and glutamine was significantly increased, and the concentration of total creatinine significantly decreased after TBI. The authors found that α-glucose was a stronger predictor cerebral metabolic rate of oxygen, increased intracranial pressure and Glasgow Outcome Scale–Extended. Taking into account the increase in propylene glycol these results, suggest changes in glucose metabolism after TBI (Glenn et al., 2013). In a later work, Orešič et al. (2016) performed a metabolomic analysis based on two-dimensional gas chromatography coupled to time-of-flight mass spectrometry of serum and brain microdialysate from patients with acute TBI (12 h). The study was focused on two groups of patients with similar TBI but from different regions—Finland and United Kingdom. The authors found an increase in the blood serum of two medium-chain fatty acids (decanoic and octanoic acids) and sugar derivatives, most of which were also found in high concentrations in brain microdialysis of patients with TBI. The authors concluded that the serum metabolites were sensitive to the severity of TBI and predicted patient outcomes (Orešič et al., 2016).

In another study Wu et al. (2016) applied a metabolomic profiling method using a differential chemical isotope labeling liquid chromatography mass spectrometry with a universal metabolome standard. CSF and sera samples from 30 patients were obtained at 3 time points (∼24, 48 and 72 h) after SCI. There were 6 CSF metabolites (uridine, imidazoleacetic acid, methionine sulfoxide, arginine, cystathionine, and homocarnosine) and 4 serum metabolites (uridine, 4-hydroxyproline, N1, N12-diacetylspermine, and glycylproline), the level of which correlated with the severity of SCI AIS A, B and C. Metabolic pathway analysis revealed a predominant dysregulation of arginine-proline metabolism after SCI (Wu et al., 2016). In a more recent pilot study by Singh et al. (2018) the correlation between the metabolic profile in the blood serum of patients with SCI and neurological recovery was analyzed using proton nuclear magnetic resonance spectroscopic. It was found that significant differences in metabolites between SCI and the control group were characteristic of 15 metabolites, of which 7 were statistically significantly different and belonged to two classes of organic compounds: amino acids (valine, isoleucine, glycine), ketone bodies (acetone, succinate, acetate) and lactate (Singh et al., 2018).

Thus, the possibility of using metabolomic profiling of CSF and blood serum in neurotrauma to study pathophysiological processes and search for biomarkers that predict disease outcomes is being considered. Using the analysis of the metabolomic profile, it is possible to draw a conclusion about which of the metabolic pathways is impaired and where therapy should be directed. However, the number of such studies is not large and requires continued work in this direction with the inclusion of a larger sample of patients in order to effectively search for reliable biomarkers. The metabolites that have already been identified in TBI and SCI are very diverse, and were found in studies using different measurement methods, which does not allow comparison of the results. In addition, in order to develop a panel of biomarkers for use in diagnostics, the absolute amount of a particular metabolite must first be established, and then analysis should be carried out in larger samples and control populations.

## Conclusion

The multicomponent nature of the processes that occurs during neurotrauma and forms complex microenvironment is the main barrier in the development of effective diagnostic and prognostic tools. In addition, dynamic post-traumatic processes and existing differences between the types of injury (for example, contusion, crush, etc.) as well as their location are also critically important for the search for potential biomarkers of neurotrauma. On the other hand, high sensitivity of components to changes in external factors (low robustness) of the most accessible and widely used ELISA methods adds complexity to the interpretation and requires validation of the results obtained. In this regard, mass spectrometric analysis methods, primarily based on MRM technology, are increasingly being used in clinical practice to improve the detection selectivity of target biomarker proteins, the search for which in neurotrauma, however, has not yet been completed. To include transcriptomic analysis in the arsenal of laboratory diagnostics, apparently, it will take even more time, which is necessary to establish a reliable picture of gene regulation and their influence on specific links of pathogenesis. When solving the existing technical problems associated with sample preparation and the features of various devices, as well as increasing the interlaboratory reproducibility of results and objectivity of the data obtained, the possibility of creating in the future ready-made panels/chips for sequencing or sets of specific primers for the detection of diagnostic transcripts in TBI and SCI is not ruled out. Given the above, it is worth noting that, before choosing the most effective and clinically feasible approach, it is necessary to carry out important work to establish common analytical protocols for determining diagnostic and prognostic biomolecules, since the results of research work available to date are often not comparable with each other and have low reproducibility. Unfortunately, we cannot fully compare the technologies mentioned in our review, since each of them is suitable for a specific task. It is possible to analyze the presented technologies only in terms of cost and turn-around time, but not the result. The detected biomolecules belong to different classes and, accordingly, have different diagnostic and prognostic capabilities in neurotrauma.
